# Factors stimulating value micro-businesses attribute to digital marketing technology (DMT) adoption

**DOI:** 10.1371/journal.pone.0260145

**Published:** 2021-12-02

**Authors:** Sunday C. Eze, Vera C. Chinedu-Eze, Hart O. Awa, Rami Hashem E. Alharthi

**Affiliations:** 1 Department of Business Studies, Landmark University, Omu-Aran, Nigeria; 2 Department of Agric Business, Michael Okpara, University of Agriculture, Umudike, Nigeria; 3 Department of Marketing, University of Port-Harcourt, Port Harcourt, Nigeria; 4 Department of Financial and Administrative Sciences, Ranyah University College, Taif University, Taif, Saudi Arabia; University of Salento, ITALY

## Abstract

Most micro-business managers in Nigeria do not see the adoption of digital marketing technology (DMT) as vital for business. Many consider it as a precondition to support managerial or operational activities, not as a tactical and/or strategic tool. Although most studies focused on large organisations, the outcome of such research may not be appropriate to micro-businesses. This is informed by the negligence of micro-businesses’ idiosyncrasies and their thought of digitalization as a precondition for managerial activities without considering the value small businesses attached to these devices in terms of aiding the use as strategic tools. This renders micro-businesses’ digitalization an under-reflected phenomenon. Yet, studies spend less on examining the factors that specifically stimulate the value micro-businesses attach to these applications, leading to constant adoption and usage. Hence, there is a need for a thorough exploration of the factors that shape the value of digital marketing applications in micro-businesses in Nigeria. The study is qualitative in nature and interviews (unstructured and semi-structured) were carried out with 26 micro-businesses which was drawn purposefully from the online database and underpinned by Technology, Organisation and Environment (TOE) framework. The study revealed eleven (11) critical success factors stimulating value micro-businesses attribute to digital marketing technology (DMT) adoption. These factors include long-term functional capacity, integration capacity, expansion capacity which are related to *technology context*. Collective capability, collaborative experience are linked to the *organisation context* while adaptive training, service delivery, customer fulfilment are linked to *environmental context*. The study also unveiled ***expectancy context*** which is linked to budget, growth and profitability and aid in the extension of the TOE framework. This study will be of importance to academics and practitioners because it provides further awareness into DMT adoption framework, factors critical to the DMT adoption and may assist in reducing the number of resources spent in search of information aimed at helping DMT adoption by micro-businesses.

## Introduction

In the contemporary knowledge economy, the increasing use of digital media by consumers has emerged as one of the most pragmatic IT innovations that drives digital marketing (DM) and business growth [1–3]. The philosophy of digital and interactive marketing spans building competitive advantage in the contexts of meeting customers’ needs, creating customer values, and strengthening developer-customer contacts and interactional ties via digital distribution channels [4–6]. DM deals with marketing activities across the internet and non-internet based digital distribution channels; it involves the use of a range of interactive technologies or digital platforms such as internet, e-mail and wireless media to connect businesses to their customers, and to market or render service to customers. Digital marketing buzzes speedily to the grass-root since the last two decades, given the viral spread of internet technology and its diverse uses, as well as mobile telephone apps [7– 9] thus, posing opportunity to leverage upon to build regular interactions and real-time knowledge sharing in the value-chain. The digital marketing technology (DMT) drives direct marketing and brings customers closer to interface with the sellers and make online orders via internet and mobile telephone apps without physical contacts or having to pass through the traditional intermediaries.

Such activities offer small businesses a level-playing ground and opportunity to engage in export for survival, and to maintain cost-effective global reach, as well as event-driven direct relationship and integration amongst trading partners [10–12]. DM produces positive impacts on export performance when used as a competitive weapon of integration to build customer satisfaction through superior product-delivery attributes [8, 13, 14]. Scholars posit that DM strategies potentially improve the performance of micro-businesses [15, 16], and assure increase in product acceptance and diffusion, cross-selling, and favourable word-of-mouth publicity [17, 18]. Some studies [13, 19, 20] affirm that the socio-economic potentials and the across board use of IT innovations, including DM, by different categories of organizations to build and/or strengthen competitive advantage and manoeuvrability inform the huge returns accruing to vendors, especially in developed worlds where internet savvy is highest. Others [21, 22] opine that the market saturation of large organizations for most technology applications stimulates IT vendors’ divestiture into other market segments, including small businesses, given that small businesses’ flexibility and aggression for globalization suggest that they supposedly more strategic targets for vendors.

Further, given that modern technologies and IT innovations bridge the dichotomies of operating capabilities and gaps between different categories of businesses; most micro, small and medium enterprises (MSMEs) leverage on digital marketing (DM) to speed up their operational development and growth [13, 23, 24]. This is common through the use of unattainable global resources to converse and carryout business activities with new and incumbent clients in a manner that integrates [3, 25, 26] and grows them in the client loyalty ladder [27, 28]. Ideally, MSMEs supposedly need modern IT-innovations (in particular DM) to strengthen collaboration and real-time inter-and intra-firm information sharing, given their socio-economic roles and their financial difficulties [3, 22, 23]. Whereas majority of large organizations implement DM applications seamlessly because their business models and operations rely on the emergence of digital technologies [29], MSMEs are uncertain about the benefits and how, when, and to which extent to leverage from digital technologies [19]. In many economies, adoption of modern technologies amongst MSMEs is accelerated by government support programmes through policy initiatives, like funding programmes and/or innovation centres [30].

Regardless of such supports and the onerous features of technology innovations, low penetration of DM amongst MSMEs remains overwhelming when compared with those of large organizations. In Nigeria and many other developing countries, most small businesses devote limited time to DMT [31] because of their (the businesses) short-term planning horizons and need for instant gains, either in terms of cost reduction or sales increase [32]. According to [3] limited resources coupled with the lack of skill and underlying network infrastructure, trusts, and security explain why most MSMEs traditionally respond to environment with software that have short-run or immediate benefits. Scholars posit that the ‘maturity models’ and approaches to assessing ‘digital readiness’ identify and highlight the best practices mostly in large organizations with little emphasis on small business context; thus, almost suggesting that MSMEs’ digitalization is an under-reflected phenomenon [33] even in the contemporary world of rapid surge in websites and online interactions within the value-chain [24]. According to [34, 35], it is an absurd to think that digitalization is a ‘black box’ to which every class of companies should adopt, given that the outcomes of research from large organizations rarely capture appropriately MSMEs’ ‘idiosyncratic givens’ needed to explain and predict their adoption behaviour.

Even though MSMEs are known for faster decisions than large organizations [24], and their adoption behaviour is often driven by environmental demands [3, 5, 6, 36] (recognize that they view adoption of DMT in terms of its support to organizational activities, rather than as a strategic tool to boost competitive advantage; hence they make little use of DMT. Similarly, the surge of internet use in Nigeria since the last decade and the huge government encouragement to MSMEs to adopt IT-innovations, turns DM adoption an opportunity to researchers and practitioners. Given that MSME managers, especially those operating in turbulent and competitive environments, desire informed decisions; it is imperative that their scepticism over robust adoption of modern technologies, including DMT, is based on the dearth of scholarly works that ally their fears and doubts. The MSMEs need to be guided to understand the value of DM in the context its level of confidence and significance, and the factors that shape such values, given that [37] adoption of DMT critically shapes business experiences. Therefore, this study provides a thorough exploration of the factors that hinder and/or facilitate the value small businesses attribute to DM applications with a view to formulate informed strategies and/or tactics. The paper is structured as follows: review of literature and proposition of theoretical model, methods, techniques and findings, as well as proposed conceptual framework and conclusion.

### Literature review

Digital marketing is referred to as marketing campaign that involves the use of electronic devices (internet and other forms of digital interaction) to promotion messages. It is a nascent aspect of marketing that uses the internet and other forms of digital communication, such as computers, tablets, mobile telephones (with WhatsApp, Instagram, Google, Twitter, Facebook, Messenger, YouTube, Zoom, and other apps.), e-mails, content marketing, and other digital media and platforms to present and promote goods and services. It may take many other channels, including online video, search engine marketing, web-based display ads, social media posts, text and multimedia messages, and any other forms of digital media. Thus, in order to differentiate from online marketing, digital marketing includes channels that do not require the use of internet, such as television, mobile telephones (in terms of SMS and MMS), call-back, and on-hold mobile ring tones. The monorail and turbo-charged speed with which people, especially those in the developed worlds, go online daily provides the marketing man with opportunities to constantly leverage on digital world and digital strategy for web-based advertising and brand image building, as well as for providing great customer experience that attracts more potential customers. However, the growth and expansion of the micro-businesses is critical to socio-economic growth of any nation [38, 39].

Micro businesses employ 1–10 persons [40] in many nations, the sector is progressively vital for creating and developing dynamic and knowledge economy. They are known for their capacity to encourage entrepreneurial skills, their flexibility and innovation, and their capability to adapt faster to changing market situations. Further, despite being a major employer of labour, contributing meaningfully to most countries’ GDP; micro-business sector in the developing economies has not fully embraced DMT and other IT-innovations. This remains obvious regardless of the successive governments’ encouragements to their growth through the adoption of new technologies needed to make them viable in a highly volatile business setting [24, 27, 41] Supposedly, micro-businesses that structure their marketing activities and policies into a culture of DM leverage on strategies and plans that create automated interaction and consciousness with their clients to win their trust [31] though majority of them are yet to establish such consciousness to a level of shaping purchase decisions [39, 40, 42, 43] While micro-business sector represents the mainstay of economies with higher income, it is not same with developing economies such as Nigeria where innovators are bedevilled with low income per-head and limited finances, high administrative costs, high collateral requirements, negative attitude to lend to micro-businesses, corruption, and unstable government policies.

According to Amirkhanpour et al., [25] access to finance is key to setting up enabling environment to reduce unemployment via the development of micro-businesses, promotion of innovation and growth of GDP. The focus of this paper is the micro-business sector particularly the service-oriented businesses, given that [33] and [44] observe that Nigeria, like many other economies, is supposedly driven by services and DM. T-O-E theoretical framework underpins the paper because apart from being classic, it is widely adopted by firms to explore what digital marketing platforms are more likely to improve firms’ values, given the influences of technology, organization, and environment contexts [24, 45]. The framework represents a vital analytical tool that unveils the essential features and drivers of innovation [33, 46] (that may shape small businesses’ values with regards to the implementation of DMT and other conservational technologies. Besides, the T-O-E framework earning robust cross-context theoretical and empirical validations [24, 47, 48] and drawing on the context of environment overlooked by Rogers’ IDT and other adoption theories, it shows realistic flexibility in the justification of technology adoption [49, 50]

Further, T-O-E framework describes intra-firm innovation adoption when compared to other frameworks [47, 51] thus, making it one of the most robust theoretical frameworks [52]. The framework permits a pool of generic factors that assist in finding solutions to myriad of phenomena investigated. Significant parts of the firm are technology, which explains tools and procedures associated with internal and external technologies, as well as the know-hows [49, 50] organization: structures, degree of monopoly, size of business and resources [47, 52] and environment: competition, macroeconomic issues and regulations [15]. Regardless of the growing scholarly prowess of T-O-E framework in the IS field and given that nothing remains same overtime, scholars [24] glamour for improving T-O-E’s lenses via context extension and theory integration. Eze et al., [53] berated [54] for proposing a specified model rather using the T-O-E taxonomy to categorize factors into relevant contexts. However because this paper is exploratory, the codes and themes in the analysis will provide us with typical extension(s) of the contexts in order to provide a more substantial analytical utility for micro-businesses.

## Methods

Majority of IT adoption studies test theories via confirmatory technique. Williams *et al*. [55] posit that nearly 65 per cent of such studies take deductive and objective approach while such other approaches as case study, inductive and interview, sentimental analysis, mathematical modelling, and thematic analysis are not commonly used. In order to provide more detailed subjective and in-depth narratives instead of statistical generalization, this study applies qualitative and inductive method. However because the study is exploratory, purposive sampling was used to discover micro-businesses’ actual opinions and experiences on the subject matter, and the units of analysis were micro-businesses at a distinct (individual) level. The focus is Owerri-North, Imo State, Nigeria, where micro-business managers were drawn from the service sector. Specifically, the participants cover those micro-businesses that have adopted one or more digital marketing devices in the last two years. Owerri was selected because she has about 1.2 million MSMEs listed in Corporate Affairs Commission, and they generate employment to about 858,003 (2.48 per cent) people [56]. The National Bureau of Statistics [56] reports that the states that have the highest number of MSMEs in Nigeria are Lagos, Oyo and Osun with 11.5 percent, 8.4 percent, and 4.1 percent respectively.

However, the states that are doing so well include Kwara, Nasarawa and Jigawa with 526.5 percent, 132.5 percent and 116 percent respectively [40]. According to Eze *et al*., [40] micro-businesses in Owerri are performing less than expected due to lack of understanding of the values of digital marketing and how it can fit into the present-day trends in the marketing system. Most small businesses in Owerri rarely have plans, guides, models or frameworks that may assist them to develop strategies to adopt DM regularly despite the number of people involved in the business. The much they do with DM is to send mails and acquire information; thus, the paper provides adoption roadmap and reinvigorates guides to future studies while helping MSMEs to know the imports of using digital marketing on daily basis. The study population includes managers and employees of selected micro-businesses drawn from Owerri Commercial Directories; of the 120 qualified participants, 26 agreed to be interviewed.

### Interview

Interviews were conducted in two stages; the first stage involved unstructured interviews with 4 participants at the preliminary level. The essence of this initial interview was to determine the present state of digital marketing in micro-businesses by analysing the interview outcome and applying samples of the raw data from the analysis to theoretical codes of technology, organization and environment to ensure that these codes fit into the subsequent raw data generated from the semi-structured interviews. This process facilitated the design of questions for the semi-structured interviews used at the second stage of the data collection. In general, 4 (V1 to V4) unstructured interviews were carried out at the preliminary stage in Table 1, while semi-structured interviews were carried out with 22 (V5 to V26) participants as depicted in Table 2. Before the interview process started, a written consent was granted by the Landmark University Review Board. A formal letters were sent to the participants detailing the purpose of the interview and assurance of confidentiality. The essence was to ensure that participants are comfortable before going through the interview. This is an important part of the interview protocol because one cannot rely on memory otherwise it may lead to error and/or bias (Oates, 2006). This process also helped the participants to determine the credibility of the research process before the commencement of the interview. The interviews lasted between 1 hour and 1 hour, 30 minutes. The study was carried out from February 2020 to October 2020.

**Table 1:** Preliminary study participants and the company profile.

Participants	Role	Company size	Sector
V1	Director	8	Telecommunications
V2	Director	9	IT software development
V3	Director	10	Telecommunications
V4	Director	9	Security Operative

**Table 2:** Major study participants and the company profile.

Participants	Role	Company size	Sector
V5	Manager	10	Finance
V6	Director	8	Finance
V7	Director	10	Security
V8	Chief Executive Officer	9	Training services
V9	Director	6	Finance
V10	Manager	9	Property development
V11	Manager	5	Wireless communication
V12	IT Manager	6	Business Consultant
V13	Manager	10	Web Marketing
V14	Director	10	Property development
V15	Director	9	Business Consultant
V16	Owner Manager	5	Finance management
V17	Owner Manager	1	Internet marketing
V18	Analyst manager	4	Finance
V19	Business Development Manager	5	Training
V20	IT manager	10	IT vendor
V21	Owner manager	6	Property management
V22	Manager	8	Property management
V23	CEO	10	Business Consultancy
V24	CEO	5	Business Consultancy
V25	Manager	7	Financial management
V26	Manager	8	Digital marketing consultant

### Data analysis

**Fig 1:**
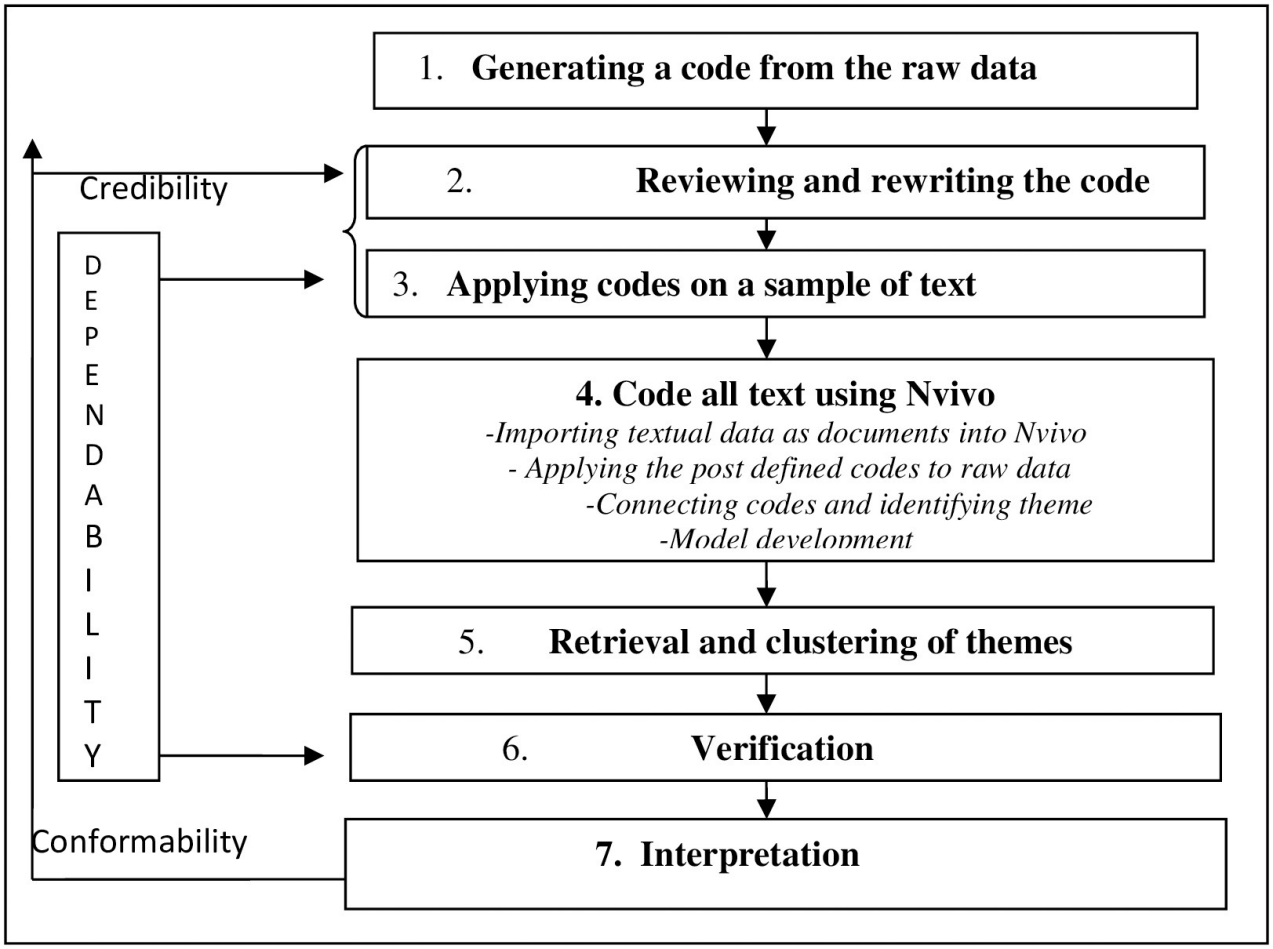
Data analysis process is about here. Source: Adapted from Eze et al., 2019.

The study adopted a theory-driven thematic analysis. This approach was adopted because the codes were obtained from the T-O-E framework while impact emerged empirically. To understand the features of each code in details the names and descriptions of the codes were highlighted in Table 3 which assisted in coding and description of data. The data analysis process in Fig 1, as a part of research design depicts a step by step approach showing how the raw data was reported (dependability check) and the conformability checks that depicts that the data is linked to the interpretation [57]. The analysis involved: (1) analysing the initial raw data from the unstructured interview in order to ascertain how suitable and applicable the theoretical codes and the empirical codes align with the raw data; (2) the use of NVivo software to manage the interview data and to help the analysis because of the huge data involved; (3) retrieval of data from NVivo and clustering them into appropriate categories; and (4) inter-coder reliability test with two judges who purified the data.

**Table 3:** Description of codes.

Technology	Technology context encapsulates internal and external variables that impact on the values micro-businesses attach to adoption of DMT.
Organization	Organization context extends to firm’s assets that influence the values micro-businesses attach to DMT adoption.
Environment	Environmental context extends to internal and external factors that shape the value micro-businesses attach to DMT adoption.
Expectancy	Expectancy refers to the anticipated benefits that shape the value micro-businesses attach to DMT.

The judges compared the codes with the themes that emerged using percentage agreement [58] (and the results as shown in Table 4 indicated that the first judge accounted for 83 per cent agreement and the second was 82 per cent; thus, exceeding 70 per cent benchmark as proposed by [59]. It is important to note that during the preliminary interview and analysis, the code *expectancy* which referred to the anticipated benefits that shapes the value micro businesses attached to DMT constantly re-occurred. This was the basis for its inclusion in the subsequent interviews and analysis.

**Table 4:** Reliability.

Areas	Number of judges	Reliability	
		First Judge	Second Judge
Factors that shape the values micro-business attached to digital marketing	2	0.83(83%)	0.82(82%)

## Findings and discussion

The tables below represent the findings of the study. It shows the categories/codes and the associated themes that emerged as a result of the analysis and their supporting cases and supporting evidence. The themes were clustered based on the features of each theoretical codes. This process has shown the factors that shape the values micro-businesses attach to digital marketing. However to aid the understanding of DM adoption in micro-business context, the interview exercise unearths expectancy context and tends to extend the T-O-E framework as shown in Table 5 below. This extension is imperative because micro-businesses do not have strong capital base and so prefer technologies that promise faster growth potential and short-term profitability amidst competition.

**Table 5:** Factors, themes and supporting cases.

Factors shaping DMT adoption	Samples of supporting evidence	Related cases	No of cases coded
**Category 1: Technology & themes**			
Long-term functional capacity	"[––] we look at how efficient digital marketing will be. Will it provide same services when it is compared with large establishments on a long range?” If it does, we will attach greater value to it and use it (V5). “The value we attach to digital marketing depends on the extent it moves our firm forward” (V2).	V1***,V3*** V2**,V5****, V7***, V9**, V11**, V14***, V15**	9
Integration capacity	“A high value is attached to any marketing tool that will fit into what we have already without much difficulty” (V3). Can this technology easily interface with other extant technologies we have at present” (V13)? "Is there any way the device can be designed to work with what we have presently? It is easier to interface?” (V15).	V1**, V2*, V3**, V10***, V13*, V14***,V15***, V16*, V17*, V20**	10
Expansion capacity	"I think in our organization, it will get to a point where we will decide to replicate and make the IT bigger". We always ask ourselves this question: can the capacity be expanded (V12). "Sure! We will adapt to applications that is expandable. If the application can accommodate existing ones we will try it (V14).	V2**, V3**,V4*, V12**, V11*** V14**,V18***,V19**,V20***,V21***, V24***	11
*Cumulative*	V1, V2, V3, V4, V5, V7, V9, V10, V11, V12, V13, V14, V16, V18, V19, V20, V21, V24		18/26 (70%)
**Category 2: Organization & themes**			
Collective capability	“As a business, we value new application more when all our staff come together to evaluate if it can help us achieve our collective objectives. Ones we ascertain this, we can either adapt or ignore it. However, most times we try the product" (V19). Whatever decisions are made here, they are the fallout of our collective effort and everybody tend to value it because we are all involved (V23).	V1***, V5****, V6***, V7***, V8**,V11**,V14*, V15**, VV19**, M22***, V23**, V24**,V26****	13
Collaborative experience	"What we have started doing now is to partner with businesses concerning these devices. When small businesses that are engaged in a similar line of business are involved in acquiring the product, the value we attach to such product increases."(V12). “Every staff in the organisation played a major role in ensuring that the decision we made about the devices are loved by customers. This also determines the level of value we place on the technology" (V15).	V1***,V2**, V3**,* V4**, V5*,V9**, V12***, V13**, V15**, V22**, V23***	11
*Cumulative*	*V1*,*V2*,*V3*,*V4*,*V5*,*V6*,*V7*,*V8*,*V9*,*V11*,*V12*,*V13*,*V14*, *V15*,*V17*,*V19*,*V22*,*V23*,*V24*, *V26*,		20/26 (76%)
**Category 3: Environment & themes**			
Adaptive Training	“We will value an application that we can easily adapt after the training. We don’t want to continue to do training all the time (V11). "How much training is required to get used to the technology? If the training cannot aid easy adaptability of the device we may not value the product and may not use it" (V2). “We must ensure that the application is always easy to learn at the initial stage” (V9).	V1**,V2**,V5**,V9*,V11**,13***,V14***,V17***,V18***,V20*	10
Service delivery	“[––] How fast is the device at all times? [—I have to sincerely tell you that how fast and consistent the device will determine the adoption rate and how we value it. (V15). The technology must have the capacity to improve our daily process and delivery “(V12). If not, we will place less value to it.	V2**,V3**, V7***, V8**, V10**, V11**, V9**, V12*, V13***, V15***	10
Customer Fulfilment	“Our customers are the king. They are the main reason we are still in business. Any application that aid in the fulfilment of customers will be highly valued and adopted. There are no two ways about it. The more it satisfies customers’ needs, the more we value it and use it. (V11). "Digital marketing technology is a means to an end. If it can help us meet the expectations of customers, it will be tried” (V12).	V1*,V4**, V5***,V6***, V7***, V10***, V12**, V14***,V16***, V23*, V24**, V25**, V26***	12
*Cumulative*	*V1*,*V2*,*V3*,*V4*,*M5*,*V6*,*V7*,*V8*,*V9*,*V10*,*V11*, *V12*,*V13*, *V14*,*V16*,*V17*,*V18*,*V23*,*V24*,*V25*,*V26*		21/26 (80%)
**Category 4 Expectancy & themes**			
Budget	"You know we are relatively small. We have to look at our budget to see if it will accommodate the new application. How cost-effective is the technology given our budget? If the application is cost-effective and can accommodate our financial plan, we are likely to value it and try it more often” (V1). “[––] Budget is one of the things we consider [––]. We will value the application more if it can help us reduce our budget in terms of cost and workforce (V15). “We must consider the budget” (V20).	V1***,V2**V3*,V5****,V6****,V9**,V11****,V1*,V15***,V16***,V20***,V2**,	12
Growth	“The primary objectives of any business is to grow continually. How often will the application assist us in increasing the number of clients we have? (V14). We don’t play with any application that we help us do that. “Can the application help us build or increase our customer base? If it can, we do not have any choice than to acquire it” (V5).	V5***,V6**, V9***, V14**, V17**, V20**	6
Profitability	“[––] We would always value applications that can help us reduce the workforce and maximize profit” (M20). “Assuming we bought the application #20,000, and at the end of the years we make #15,000 profit we will adopt it (V23).	V2*, V5***, V9**, V12***, V13***, V14***, V20**, V23**	8
*Cumulative*	*V1*,*V2*,*V3*,*V5*,*V6*,*V9*,*V11*,*V12*,*V13*,*V14*,*V15*,*V16*,*V17*,*V20*,*V23*		15/25 (60%)

Note: V1, V2….V26 signify the respondents, while *signifies the number of times V1 to V26 referred on a text.

### The framework

**Fig 2:**
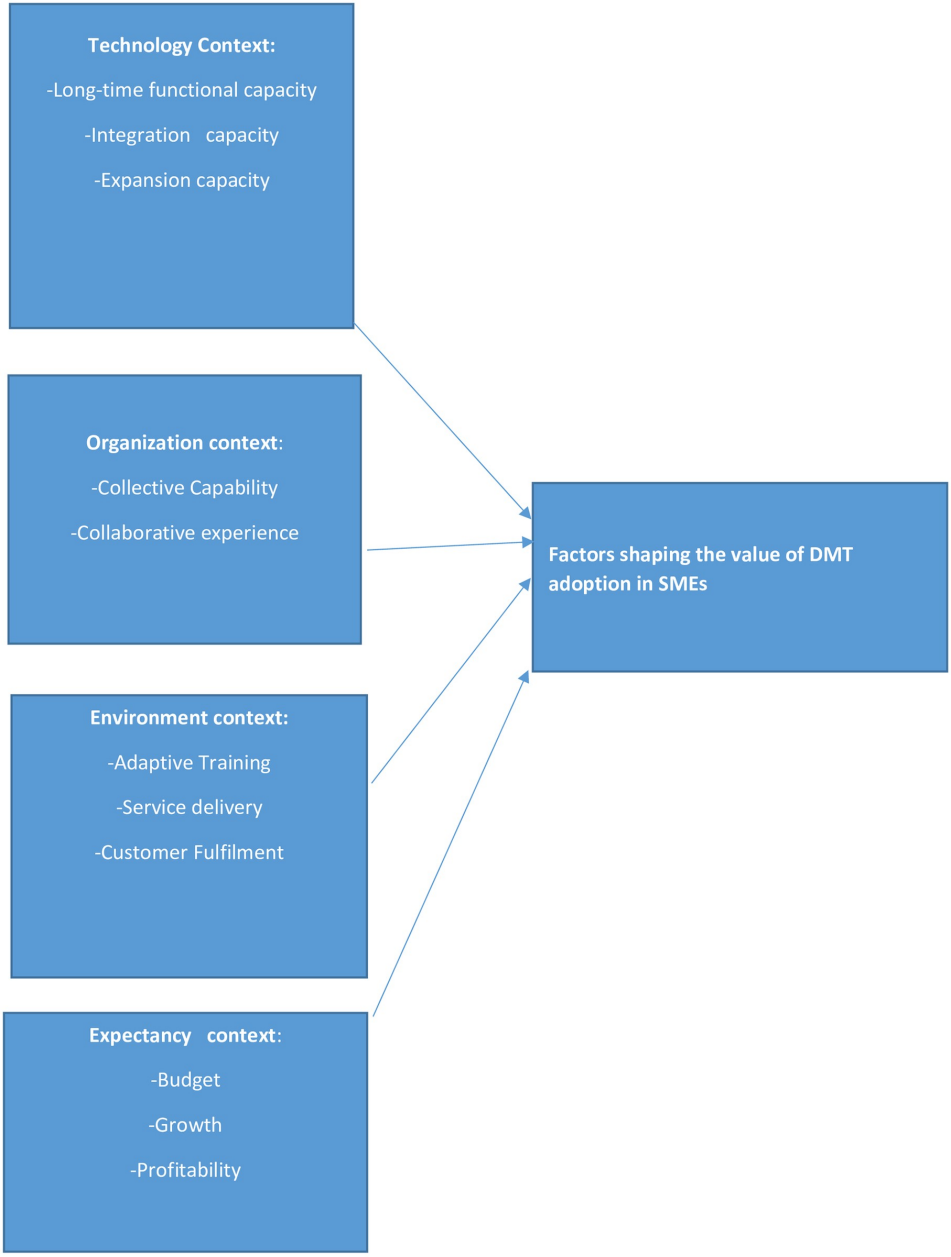
Factors stimulating values micro-businesses attach to digital marketing devices is about here.

Fig 2 below depicts a conceptual framework for the study showing the factors that shape the values micro-businesses attach to digital marketing devices. These factors were explored based on theory-driven thematic analysis using -*Technology*, *organisation and environment* and the data-driven one—*expectancy*, which emerged empirically to represent the *anticipated benefit attached to the digital marketing by SMEs*. The exploratory and the explanatory of nature of the finding in the framework are based on the participant’s own narratives and literature. This helps in the explanation of the factors. Although the study is of the view that the developed framework may serve as a frame of references to the understanding of the factors that may shape the values MSMEs attached to digital marketing devices, the meanings of the codes derived operationally and the factors could be adopted as analytical instruments for researchers to understand and explain factors that continually shape the values of digital marketing applications in MSMEs.

While the adoption of digital marketing applications may help micro-businesses to create partnerships and develop new business ideas to enhance information sharing, collaborative environments and advanced product and process innovation [5, 60, 61] however, one of the striking findings is the emergence of the *expectancy context* which aid in the extending the framework. Drawing on the finding, although the majority of micro-businesses may have the zeal to exploit opportunities using these applications to support fast decision-making [5, 62] small businesses do not often have the zeal to try new technologies. Hence, the finding discussed below may play an important part to small businesses in valuing these applications and become hand-on and tactical in dealing with DMT in the future.

### Technology

The first construct, long-term functional capacity, defines the extent to which DMT helps the business to meet its long-run obligations. Small businesses have financial limitations as they struggle to remain commercially viable and sustainable. Hence, the adoption of DMTs may not be easy although it is comparatively cheap [63–66]. Evidence from the analysis shows that micro-businesses would value applications that accomplish tasks in a long-run without complexity as raised by [V5], [V11], [V14], [V15] [V2], [V7] and other interviewees to show cross-case supports (see Table 5). Previous [67–70] and recent [47, 50, 71, 72] studies support this finding when they reported that digital devices that are complicated, lack functional capability, and slow business processes rarely stand the test of time. Second, integration capacity defines the extent to which DM devices consistently adapt to incumbent technology used by the firm, given that [73] posit that inconsistency reduces when an application is capable of integrating into extant arrangement. MSMEs tend to value DM applications that seamlessly adapt to what already exists in the organization as echoed by participants (V1, V2, V10, V13, V14) and their cross-case supporters (see Table 5).

Studies [74–77] affirm that it may be expensive to have new applications whose features do not fit into, or integrate, the old and current infrastructural arrangements. Micro-businesses rarely value applications that do not easily adapt to the organization’s norms. Third, expansion capacity defines the ability of digital marketing device to continually accommodate and integrate emerging and extant technological capabilities [33, 66, 78]. The analysis shows that micro businesses place significant values to marketing devices that are flexible and can constantly intertwine with new and emerging features. It was noted that the expansion nature of any digital marketing devices aid in business and process innovation. It controls the cost of engaging in entirely new technology. Narratives from 11 interviewees [V14; V2; V3; V4; V12; V11; V18; V19; V21; V24; V20] severally affirm that micro-businesses place high value on those marketing devices that easily integrate with other ones (see their comments in Table 5). Scholars [71, 77, 79, 80] (reported that small businesses attach higher value to IT applications that adapt to extant organizational norms.

### Organization

Collective capability is the capacity of small businesses to understand and accommodate the views and initiatives of the majority of the workforce via open interaction. It is argued that the views of micro business managers on digital marketing may not be the same with those of others based on differences in knowledge acquisition and social setting. [V19] and [V23] with many cross-case supporters {[V1]; [V5]; [V6]; [V7]; [V8]; [V11]; [V14]; [V15]; [V19]; [V22]; [V23]; [V24]; [V26]} reported that understanding and integrating everyone’s views help to disseminate technical information that smoothens relationship. Gbadegeshin *et al*., [71] and, Nelson and Cooprider [81] found that when information is understood by all, it helps to improve efficiency and bring about collective value and understanding. Shared meaning is vital and how it circulates across the various levels in an organization shapes the values attached to technology [71, 82]

Often businesses partner extensively to build and leverage on integrated competitive advantages. This leads to collaborative experience, which represents the extent to which integrated businesses are committed to collective problem-solving in order to benefit from comparative cost advantages. However, the finding reveals that most small-businesses do not collaborate when the IT-applications rarely help them to understand their trading partners. This implies that more value is placed on digital marketing devices when it aid collaboration among workgroups as was noted by participants [V15], [V22], [V23] and cross-case supporter (see Table 5): [V1], [V2], [V3], [V4], [V5], [V9], [V12], [V13]. This was consistent with previous studies [71, 78, 83–86] that found that corporate initiatives and collaborative efforts aid IT-adoption decision.

### Environment

Often technology regularly evolves and regularly turns obsolete. First, adaptive training defines the ability of the manager to swiftly learn and adapt to the training required to operate a new technology. Adaptive training is done to help owners and workers to learn on how DMT adds values to business developments. This study discovered that small businesses tend to value new applications that are simple and allow ease of use without having to undergo a series of rigorous training. Participants [V11; V2; and V9] and cross-case supporter [V1, V5, V9, V13, V14, V17, V18, and V20] severally and individually are of this position. According to [18] majority of MSME managers lack the patients and devotion to learn about DMT because most of them, believe that such new technology consumes their time. In other words, new applications are more valued by MSMEs when huge resources are not required to learn and understand the applications [44, 85]. Second, service delivery defines the ability of DMT to advance business activities proficiently and profitably. Participants V15 and V12 and cross-case supporters [V2 V3, V7, V8, V10, V11, V9, and V13] are of the view that DMT would be highly valued if the information circulated is consistent and dependable, improves daily processes, and helps to assist customers to make fast and real-time decisions.

This is consistent with previous studies [16, 85, 87, 88], that espouse the dependability of efficiency of service delivery in terms triggering prospective clients to turn genuine adopters. Third, given that [89] posits that customer is the king and that, he largely values new applications that help them to improve market shares and market positioning, customer fulfilment defines perceived satisfaction derived from using DM devices. Participants V11 and V12 and cross-case supporters [V1, V4, V5, V6, V7, V10, V14, V16, V23, V24, V25, and V26] recognized the criticality of the customer in the life and existence of any firm and observed loudly that any application that represents a worthy means to an end and fulfills the customer expectations is highly valued and adopted. Scholars [16, 85, 87, 89] propose that the attributes of any technology are very important in decision process and vital to the success of the promoter if they help to acquire, retain and fulfil customer’s needs.

### Expectancy

Often MSMEs are deprived of emerging technologies because of their constraints of limited resource; thence, they relate their budget to the ability of DMT to assist processes at a moderate cost. In other words, MSMEs value and appreciate more applications that are capable of minimizing cost without compromising quality. Participants [V1, V15, V20, V2, V3, V5, V6, V9, V11, V16, and V2] strengthened this position when they recognized that their small size makes them to do critical cost-benefit analysis in order to make informed decision on budgets. This consistent with previous studies [16, 47, 72, 90] who view that small businesses clinch to DMT if the cost-benefit structure is long-ranged. Firm’s growth, as the second construct, is tied to staff strength, market share, and applications that aid business processes. DM devices advance productivity and help micro-businesses to develop new industries and business activities.

This was echoed across cases [V5, V6, V9, V14, V17, and V20] when the cases recognized that adoption is valued primarily if the technology enables firm’s growth in terms of customer base. Previous studies [65, 66, 91–93] affirmed that MSMEs value digital marketing applications that have specific growth objectives and improve business performance. Finally, profitability is the return on funds invested in DMT, particularly in the views of scholars [33, 94] profitability includes unusual returns and market values. Case and cross-case participants [V2, V5, V12, V13, V9, V20, V23 and V14] note that they value applications that are capable of reducing workforce and maximizing profits. Studies show that when businesses make profits following technologies adopted, that triggers off investment because such technologies are valued [83, 95].

## Conclusion and implication

This study examined 14 factors within the extended contexts of T-O-E with a view to proposing a model that supports the understanding of the value micro-businesses attribute to DMT adoption. Drawing on the classic elements of the T-O-E framework, these factors were driven by theories and raw data. Within the technology context, long-term functional capacity, integration capacity and expansion capacity were the factors that shape the values micro-businesses attach to digital marketing. Collective capability and collaborative experience were linked to organization context, while environmental context was associated with adaptive training, service delivery and customer fulfilment. The expectancy context was linked to the budget, growth and profitability. Although these factors are deemed vital in determining the value micro-business place on digital marketing devices, the extent of value on such application varies. Implicit is that conventional theories used to explain and predict technology adoption need to extend their factors/contexts, and/or integrated to give a robust and more complete explanation. This may be particularly paramount, given that there is no distinct theory that concretely exhausts all the factors that explicitly explain MSMEs’ adoption features relating to DMT. Thence, the paper extended the T-O-E framework by integrating additional context (expectancy), as well as the factors that link to it. The expectancy context and the associated factors do not only help to extend the T-O-E framework but also offer richer explanatory lenses to understanding of the adoption behavior of MSMEs.

The extended conceptual framework appears a bit more robust, helps to explain intra-firm adoption behavior better, and provides critical analytical scope of DMT adoption. It gives a clearer direction on the specific factors that influence the values small businesses attach to adoption practices in the developing economy context. Further, the paper offers a more critical insight that recognizes the culture and socio-economic peculiarities of the developing nations, which often limit the workability of some classic adoption theories developed in the Western World.

Practically, small businesses have continually reflected on what approach to use in adopting digital marketing devices that will be of value to them in order to gain new and broader insights on how best the devices can be deployed overtime amid the complex and volatile the environment. Hence, the framework is practically important to experts and/or practitioners who make day to day business decisions and seek to develop frameworks for IT application. The proposed conceptual framework may be valuable to small businesses by assisting them to have a more in-depth insight on the factors that may shape the values they attribute to digital marketing applications. Such insight may aid MSMEs to create awareness on the need, value and use of these devices in order to avert some future challenges.

### Limitations and future research

The study is qualitative and so, the research design, rigour in data gathering and collection, management of the large raw data involved, and the analysis, as well as the credibility of the findings, may require further validation. This is because it may lead to unforeseen–respondent- research bias in the data analysis. This may result in a limited understanding of alternatives and insights into the factors shaping the values of digital marketing technology adoption. Hence, methods such as case study and mix-method may be adopted to further validate the findings. Further, the study interviewed 26 interviewees, which is relatively small; hence, the generalization of the findings and the framework may be a bit difficult in some context. It is suggested that other studies should use other approaches to test, ascertain and validate the findings and the framework across a wider population. While the study is of the view that the framework may not be suitable across countries because of the level of exposure, variances in the level of technology use, culture, socio-economic viability, further studies are needed to draw on the framework and to extend the factors.

## Supporting information

S1 Data (DOCX)
