# Supplementary material for: Factors stimulating value micro-businesses attribute to digital marketing technology (DMT) adoption
Source: PLoS One. 2021 Dec 2;16(12):e0260145. doi: 10.1371/journal.pone.0260145 (PMC8638917; doi:10.1371/journal.pone.0260145)
Supplement: S1 Data — (DOCX) [file pone.0260145.s001.docx]

| **Factors shaping DMD adoption: THEMES** | **Evidences** |
| --- | --- |
| **Category one : Technology:** | "[--] we look at how efficient digital marketing will be? Will, it provides the same services when it is compared with large establishments for a long time?” If it is, we will attach greater value to it and use it  “The value we attach to digital marketing will depend on the extent it moves the company forward  ‘Limitless location, can it reach limitless consumers it will reach’  ‘Does it creates link for relationship, quick feedbacks it is loaded with opportunities and connections’ |
| Long time functional capacity |  |
| Integration capacity | “A high value is attached to any marketing tool that will fit into what we have already without much difficulty  Can this tool easily interfaces with other tools we have presently”  I don’t want a situation where, I will have to abandon the systems  I have been using before thereby wasting money. Can it fit into what we are using presently?  I thank I will have a problem if my employees can use it what the systems we have already |
| Expansion capacity | "I think in our organization, it will get to a point where we will decide to replicate and make the IT bigger". We always ask ourselves this question-can the capacity be expanded”  “Definitely, if the system is flexible in nature, that it could accommodate improvement in future, it will be considered, and the value we attach to it will be greater”  "Sure! We will adapt to applications that is expandable. If the application can accommodate existing ones we will try it”  “A have no problem what so ever, as long as the system will have room for expansion any time and any day”.  “If the rate at which it accommodate new thing will aid the business to have more regard to it” |
| **Category 2: Organization** | “As a business, we value an application more when all our staff come together to evaluate if the new technology can help us achieve our collective objectives. Ones we ascertain this, we can either adapt or ignore it. However, most times we try the product"    “I cannot do it alone because I am not the one that we use it. We have people who are working with us. They must decide “  “Whatever decisions are made here is a collective effort and everybody tend to value it because we are all involved2  “I normally ask everyone to contribute. This alone make them have a sense of responsibility and value”  “It would be of interest to note that when two or three are gathered to take a decision, it is more valuable compared to one person taking a decision” |
| Collective capability |  |
| Collaborative experience | "What we have started doing now is to partner with businesses concerning these devices. When small businesses that are engaged in a similar line of business are involved in acquiring the product, the value will attach to such product increases”  “Every staff in the organisation played a major role in ensuring that the decision we made about the devices are loved by customers. This also determines the level of value we placed on the technology"  “ the value one has will depend on the collective experiences exhibited by members of the organization”  Experiences matters a lot. When people that have been in a company for so long and masters the act, give advices, it then to help the organization move forward and thus stimulate values people attach to the outcome. |
| **Category three: Environment** | “We will value an application that we can easily adapt after the training. We don’t want to continue to do training all the time”  "How much training is required to get used to the technology? If the training cannot aid easy adaptability of the device we may not value the product and may not use it".  “If we continue to be trained in one system over and over, we will not really value the application compared to when a little training is given to it”  “The system we value most depends on how easy we can use it after training” |
| Adaptive Training |  |
| Service delivery | “[--] How fast is the device at all times? [-- I have to sincerely tell you that how fast and consistent the device will determine the adoption rate and how we value it”  The technology must have the capacity to improve our daily process and delivery “  Can it help us in day to day business? If not, we will place less value to it.  Easy life, easy understanding less stress’ |
| Customer Fulfilment | “Our customers are the king. They are the main reason we are still in this business. Any application that aid in the fulfilment of customers will be highly valued and adopted. There are no two ways about it. The more it satisfies customers' needs, the more we value it and use it.  “You know, customers are the kings. Any application that can aid in satisfying our customers need will be our priority “  "Digital marketing technology is a means to an end. If it can help us meet the expectations of customers, it will be tried “  “Whatever we do here is because of the people that buy our product. Application that can regularly aid in retaining and acquiring new customers will be considered first “ |
| **Category four: expectancy :** | "You know we are relatively small. We have to look at our budget to see if it will accommodate the new application. How cost-effective is the technology about our budget? If the application is cost-effective and can accommodate our financial plan, we are likely to value it and try it more often”  “I am a man one business, and I look for solutions that are less expensive and also help me in competing effectively. If I get such solutions I would really appreciate it.”  “[--] Budget is one of the things we consider [--]. We will value the application more if it can help us reduce our budget in terms of cost and workforce?  “How much it cost will go a long way in helping us take a stand in valuing it”. |
| Budget |  |
| Growth | “The primary objectives of any business to grow. How often will the application assist us in increasing the number of clients we have?  “We don't play with any application that we help us do that”  “Can we application help us build or increase our customer base?    “We may not have any choice than to acquire application that can aid my business to grow. I place much emphasis on that” |
| Profitability | “[--] We would always value applications that can help us reduce the workforce and maximize profit”  “I really need to break even anytime I start something new, and would value any solution that can help me”  “I don’t care about it. Just get us what will increase our profit base”  Why I am in business? It is to make profits I will continue and value such solutions.  “Assumes we bought the application #20,000, and at the end of the years we make #15,000 profit we will adopt it |
